# Osteoradionecrosis in osseous free flaps after maxillofacial reconstruction: a single-center experience

**DOI:** 10.3389/fonc.2025.1527149

**Published:** 2025-01-30

**Authors:** Jakob Fenske, Philipp Lampert, Eirini Nikolaidou, Claudius Steffen, Marcus Beck, Norbert Neckel, Susanne Nahles, Max Heiland, Friedrich Mrosk, Steffen Koerdt, Carsten Rendenbach

**Affiliations:** ^1^ Department of Oral and Maxillofacial Surgery, Charité Universitätsmedizin Berlin, Corporate Member of Freie Universität Berlin and Humboldt-Universität zu Berlin, Berlin, Germany; ^2^ Department of Radiation Oncology, Charité Universitätsmedizin Berlin, Corporate Member of Freie Universität Berlin and Humboldt- Universität zu Berlin, Berlin, Germany

**Keywords:** mandibular reconstruction, osseous free flaps, osteoradionecrosis, adjuvant radiotherapy, fibula free flap, free flap complications, flap osteoradionecrosis

## Abstract

**Objective:**

In the multimodal treatment of advanced head and neck malignancies, primary free flap reconstruction in a one stage procedure with tumor resection is frequently combined with adjuvant radiotherapy. Radiotherapy is known to exhibit side effects on transplanted free flaps, including osteoradionecrosis (ORN) of native and transplanted bone. This study aims to evaluate the therapeutic outcomes and potential predictors of free flap ORN within osseous free flaps based on a large-scale, single-center database.

**Methods:**

A retrospective analysis was conducted on patients who underwent osseous free flap reconstruction of maxilla or mandible in a one stage procedure followed by adjuvant radiotherapy due to an advanced head and neck malignancy between April 2017 and July 2023. After case matching, patients with and without free flap ORN were assessed for potential predictors using univariate and multivariate analysis.

**Results:**

112 patients met the inclusion criteria. 21 patients (19%) developed ORN within the free flap. Following case matching, 42 patients (10 females, mean age 61.5 ± 9.1 years) were included in the final analysis. The mean time to ORN diagnosis was 19 (7–56) months after surgery. Total flap loss occurred in 7 patients (33%) following flap ORN. Smoking (76% vs. 38%; OR 5.78; p=0.03) and prior plate exposure (67% vs. 24%; OR 5.61; p=0.03) emerged as robust predictors of flap ORN in uni- and multivariate analysis.

**Conclusion:**

Osseous free flap ORN is a severe radiooncologic complication, often resulting in total flap loss and subsequently increased morbidity. Smoking and prior plate exposure were identified as key predictors of flap ORN development. Individual risk assessment and careful evaluation of osseous free flap irradiation must be evaluated in future radiooncological concepts.

## 1 Introduction

The contemporary treatment of head and neck malignancies often involves multimodal approaches, including radiotherapy combined with microvascular reconstruction using osseous free flaps. Autologous microvascular free flaps are the gold standard for reconstructing extensive defects following resection of malignant and benign tumors, trauma, osteoradionecrosis and osteomyelitis. Alternative procedures include regional flaps or osteosynthesis plates combined with local flaps, although these options compromise functionality and aesthetic outcomes. Despite constant advancements in both surgery and radiotherapy, extensive complications can still arise following the irradiation of osseous free flaps. These complications include bone exposure, osseous non-union, and, notably, osteoradionecrosis (ORN) developing within these radiovulnerable and initially healthy transplanted tissues (1, 2).

While the risk factors and potential pathomechanisms of ORN have been extensively studied in relation to the native mandible, there is a significant gap in the literature regarding its formation within osseous free flaps. Some of these studies suggest that osseous free flaps should be considered structures at risk during postoperative radiotherapy to mitigate long-term complications (2, 3). ORN formation in osseous free flaps has been observed at doses exceeding 60 Gy (4, 5); however, previous works from our group did not find elevated doses associated with free flap ORN formation. Additionally, free flap ORN have been frequently noted near patient-specific reconstruction plates (6).

Despite these observations, individual risk factors and pathomechanistic features, such as vascular parameters (7), have not been adequately identified for ORN formation in osseous free flaps. Given the initially linear blood supply via the vascular pedicle, osseous free flaps offer a suitable platform for studying these vascular parameters. Considering the complications that can arise from free flap irradiation and the associated reduction in dental rehabilitation outcomes with implants (8–10), this topic holds significant clinical relevance, particularly in the context of immediate tumor resection and reconstruction with microvascular flaps (11).

In this single-center, retrospective study, we employ a matched-pair analysis of irradiated osseous free flaps to identify individual, surgical and radiooncological risk factors associated with ORN formation.

## 2 Materials and methods

### 2.1 Study cohort

**Figure 1:**
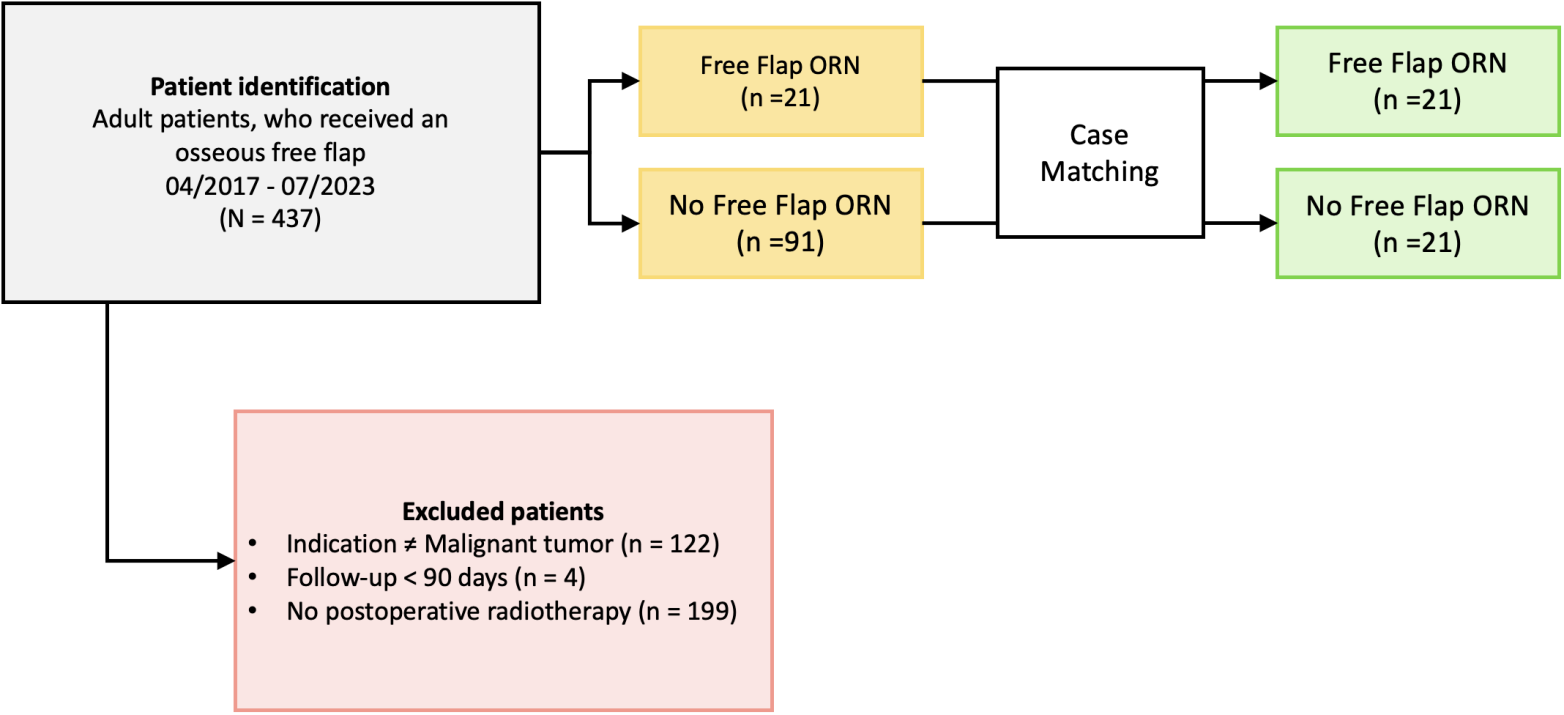
Patient inclusion and case matching process. (ORN, Osteoradionecrosis).

Ethical approval was obtained by the local ethics committee at the Charité Universitätsmedizin Berlin (EA2/077/20). Patients who received radiotherapy after osseous free flap surgery following oral cancer resection between April 2017 and July 2023 at the Department of Oral and Maxillofacial Surgery at the Charité Universitätsmedizin Berlin, Germany, were identified. The inclusion criteria were: (1) patients receiving radiotherapy to osseous flaps who were (2) at least 18 years of age at the time of surgery, (3) received a microvascular osseous free flap (fibula, scapula, iliac crest) following (4) resection of head and neck malignancies. Patients with available follow-up below 90 days after transplant surgery were excluded (**Figure 1**).

### 2.2 Outcomes and analyzed variables

The primary endpoint was the occurrence of ORN inside the transplanted osseous flap. ORN was diagnosed in previously irradiated osseous flaps showing bone exposure with or without inflammation and radiological signs of osteonecrosis. After surgical intervention, tissues were pathologically examined. Patient (sex, age, constitutional data, comorbidities), initial disease (tumor and peri-interventional specifications), and treatment characteristic (resection area, flap specifications, PORT data, dental rehabilitation with implants, complications and treatment) as well as follow-up documentations were collected until July 2024.

### 2.3 Case matching

To account for potential confounders and improve comparability, we performed a matched-pair analysis. Each patient with flap ORN was matched with an irradiated patient without flap ORN. Matching criteria, in ranked order, included reconstruction site (mandibula, maxilla), age, gender and time of follow-up. We employed a nearest neighbor matching algorithm without replacement using SPSS, Version 29 (IBM Corp.). All eligible patients were successfully matched, and the final analysis was conducted using this matched cohort.

### 2.4 Analysis of radiation treatment plans

Osseous aspects of free flaps were manually contoured for each axial slice in the planning computed tomography (CT) of the original treatment plan using the software ARIA OIS, Version 16 (Varian Medical Systems). Osteosynthesis material was excluded from the retrospectively contoured volume. General treatment specifications (fractions, total doses, treatment mode and duration) were documented. Applied mean (Dmean) and maximum doses (Dmax) to the flap and volumes exposed to 35-70 Gy (V35-70) were calculated from the dose-volume-histograms in 5 Gy steps (12, 13).

### 2.5 Statistical analysis

All statistical calculations were performed using SPSS, Version 29 (IBM Corp.). Results were considered statistically significant if the p-value was <0.05. Inclusion of the null value in the 95%-confidence interval (CI) of odds ratios (OR) was recorded as non-significant, while non-inclusion was recorded as significant. For qualitative variables, comparisons between patients with and without ORN were made using the Chi-squared test. In cases where the expected cell counts were below five, Fisher’s exact test was used instead. For quantitative variables, the distribution was first assessed for normality using the Shapiro-Wilk test. Based on the results, Mann-Whitney U test was conducted to compare means between the groups. OR and 95%-CI were subsequently calculated via binary logistic regression for quantitative variables. Parameters that showed significant differences (p < 0.05) in these univariate analyses were subsequently included in a multivariate analysis. Specifically, a binary logistic regression was performed to identify independent risk factors for the development of ORN in the osseous flaps. The discriminative ability of the model was assessed using a receiver operating characteristics (ROC) curve. The area under the curve (AUC) was calculated to evaluate the predictive performance.

## 3 Results

### 3.1 Patient characteristics

A total of 112 patients met the inclusion criteria. 21 patients (19%) presented with flap ORN. After matching, 42 patients were included and divided in two groups: irradiated patients with and without flap ORN. All analyzed patients were initially diagnosed with oral squamous cell carcinomas. The mean follow-up time was 38 (6–81) months, 10 (24%) patients were female. The mean age at surgery was 61.5 (SD 9.1) years. In univariate analysis, patients who developed flap ORN had a higher prevalence of preoperative history of smoking (76% vs. 38%; OR 5.20; 95%-CI [1.37;19.87]; p=0.01) (**Table 1**).

**Table 1:** Patient characteristics and comorbidities.

Variable	Flap ORN No. (%)	No Flap ORN No. (%)	p	OR [95%-CI]
Female	5 (24%)	5 (24%)	>0.99	1.00 [0.24;4.14]
Mean Age at surgery (SD)	62.2 (8.8)	60.7 (9.6)	0.44	1.02 [0.95;1.09]
Mean BMI (SD)	22.9 (3.8)	24.5 (5.1)	0.15	0.92 [0.80;1.06]
Mean Follow-up time in months (SD)	38.2 (21.1)	38.4 (18.7)	0.80	1.00 [0.97;1.03]
Mean hospitalization in days (SD)	20.6 (9.0)	19.7 (7.7)	0.95	1.01 [0.94;1.09]
Comorbidities				
Smoking	16 (76%)	8 (38%)	***0.01***	5.20 [1.37;19.78]
Alcohol abuse	11 (52%)	8 (33%)	0.21	2.20 [0.63;7.66]
Diabetes mellitus	2 (10%)	2 (10%)	>0.99	1.00 [0.13;7.85]
Arteriosclerosis	2 (10%)	3 (14%)	>0.99	0.63 [0.09;4.23]
Hyperlipidemia	2 (10%)	1 (5%)	>0.99	2.11 [0.18;25.17]
History of thrombosis	2 (10%)	0 (0%)	0.49	n.a.

(ORN, Osteoradionecrosis; SD, standard deviation; OR, odds ratio; CI, confidence interval; BMI, body mass index). Underlined and bold values indicate statistical significance.

### 3.2 Surgery and treatment specific parameters

Fibula, scapula and deep circumflex iliac artery free flaps were used for reconstruction. 95% (n=20) of flap ORN occurred in the mandible. Likewise, 95% (n=107) of all flaps in the initial cohort were placed in the mandible. All patients received intravenous antibiotics postoperatively for one week. If infections in the flap area occurred, local antiseptics and oral antibiotics were administered. Oral rehabilitation with dental implants did not significantly differ between both groups. In univariate analysis, patients who developed flap ORN had a higher prevalence plate exposure prior to ORN diagnosis (67% vs. 24%; OR 6.40; 95%-CI [1.65;24.77]; p=0.005) (**Table 2**). ORN occurred either in the anterior or posterior flap regions or circular around the flap segments. All ORN lesions were mainly located on the vestibular aspect of the flaps, mostly adjacent to osteosynthesis materials. Posterior ORN lesions (24%) exhibited prior plate exposure in 80% of cases and were initially covered by skin paddles in 80% of cases. Circular ORN lesions (28%) had exposed plates in 66% of cases and were initially covered by skin paddles in 83% of cases. All posterior and circular lesions occurred next to reconstruction plates. Anterior ORN lesions (48%) showed prior plate exposure in 60% of cases and were initially covered by skin paddles in 50% of cases. Anterior lesions occurred next to reconstruction plates in 50% and next to mini plates in 50% of cases.

**Table 2:** Surgery and treatment specifications.

Variable	Flap ORN No. (%)	No Flap ORN No. (%)	p	OR [95%-CI]
Reconstruction site				
Mandible	20 (95%)	20 (95%)	>0.99	1.00 [0.06;17.12]
Maxilla	1 (5%)	1 (5%)	>0.99	1.00 [0.06;17.12]
Mean surgery duration in minutes (SD)	602.5 (181.3)	574.2 (109.6)	0.44	1.00 [1.00;1.01]
Donor site				
Fibula free flap	18 (86%)	20 (95%)	0.60	0.30 [0.03;3.15]
Scapula free flap	2 (10%)	1 (5%)	>0.99	2.11 [0.18;25.17]
Deep circumflex iliac artery free flap	1 (5%)	0 (0%)	>0.99	n.a.
Intraoral skin paddle	11 (52%)	6 (29%)	0.12	2.75 [0.77;9.86]
Extraoral skin paddle	4 (20%)	2 (10%)	0.66	2.24 [0.36;13.78]
HCL classification of mandibular defects				
H	2 (10%)	1 (5%)	>0.99	2.11 [0.18;25.17]
L	4 (19%)	5 (24%)	0.71	0.75 [0.17;3.31]
C	0 (0%)	0 (0%)	n.a.	n.a.
LC	9 (43%)	6 (29%)	0.33	1.88 [0.52;6.76]
LCL	6 (28%)	9 (42%)	0.33	0.53 [0.15;1.92]
Osteosynthesis type				
Patient-specific 3D-printed reconstruction plate	15 (71%)	13 (62%)	0.51	1.54 [0.42;5.61]
Patient-specific 3D-printed mini plates	0 (0%)	2 (10%)	0.49	n.a.
Patient-specific 3D-printed reconstruction and mini plates	6 (29%)	5 (24%)	0.73	1.28 [0.32;5.09]
Handbent plates	0 (0%)	1 (4%)	>0.99	n.a.
ORN-specific risk factors				
Plate exposure	14 (67%)	5 (24%)	***0.005***	6.40 [1.65;24.77]
Plate removal	8 (38%)	(38%)	>0.99	1.00 [0.29;3.48]
Inadequate oral hygiene	6 (29%)	2 (10%)	0.24	3.80 [0.67;21.59]
Infection in flap area	11 (52%)	6 (29%)	0.12	2.75 [0.77;9.86]
Dental implant placement	2 (10%)	2 (10%)	>0.99	1.00 [0.13;7.85]
Bite marks in flap area	1 (5%)	1 (5%)	>0.99	1.00 [0.06;17.12]

(ORN, Osteoradionecrosis; SD, standard deviation; OR, odds ratio; CI, confidence interval). Underlined and bold values indicate statistical significance.

### 3.3 Radiooncological parameters

Radiotherapy (RT) was delivered as proton therapy via volumetric modulated arc therapy (VMAT) in all cases. The mean RT duration was 39.4 days (SD 7.5). The high risk area received a mean dose of 60.2 Gy (SD 4.4) in mean fractions of 2.2 Gy (SD 0.1) per day. The mean duration of RT was significantly longer in patients with flap ORN (41.86 vs. 36.86 days; p=0.04) (**Table 3**).

**Table 3:** Radiation exposure to osseous free flaps.

Variable	Flap ORN	No Flap ORN	p
Mean duration of RT in days (SD)	41.86 (6.9)	36.86 (7.5)	0.04
Mean high risk area dose in Gy (SD)	61.11 (4.5)	59.18 (4.3)	0.21
Mean fractions per day in Gy (SD)	2.16 (0.1)	2.17 (0.1)	0.52
Dmax on Free Flap in Gy (SD)	63.22 (4.8)	61.99 (4.8)	0.68
Dmean on Free Flap in Gy (SD)	59.59 (5.1)	58.07 (3.8)	0.51
Mean V35 [%] (SD)	100% (0)	100% (0)	>0.99
Mean V40 [%] (SD)	100% (0)	99.9% (0.2)	0.32
Mean V45 [%] (SD)	99.8% (0.3)	99.9% (0.4)	0.97
Mean V50 [%] (SD)	99.2% (2.0)	99.2% (2.0)	0.95
Mean V55 [%] (SD)	89.6% (18.7)	81.7% (24.9)	0.19
Mean V60 [%] (SD)	49.7% (48.9)	35.2% (44.5)	0.31
Mean V65 [%] (SD)	16.8% (31.0)	10.7% (23.4)	0.58
Mean V70 [%] (SD)	6.5% (22.9)	4.8% (21.8)	0.57
Concomitant systemic Chemotherapy	9 (43%)	7 (33%)	0.53
Anamnestic head-neck RT	1 (5%)	1 (5%)	>0.99

(ORN, Osteoradionecrosis; SD, standard deviation; Vx [%], flap volume that receives a radiation dose of x Gray; RT, Radiotherapy).

### 3.4 Therapeutic consequences of Flap ORN

**Figure 2:**
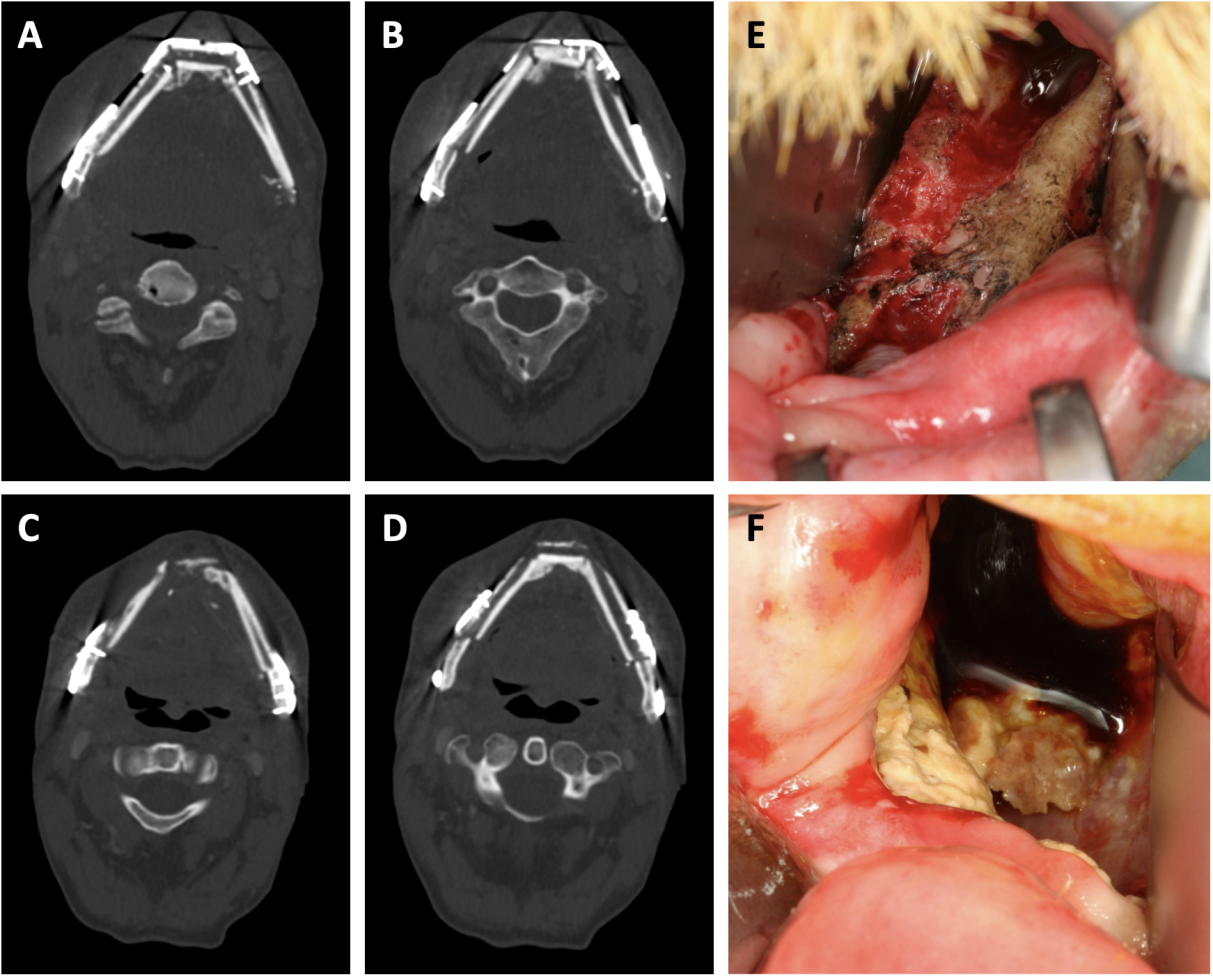
Clinical presentation of free flap osteoradionecrosis. **(A, B)** show sequences of the neomandibula prior to radiotherapy. **(C, D)** show sequences of the same transplant after partial plate removal and osteoradionecrosis diagnosis. **(E, F)** show clinical aspects of intraorally exposed necrotic bone aspects due to osteoradionecrosis.

The mean time to flap ORN diagnosis was 19 (7–56) months after surgery. Notably, 86% (n=18) of cases were diagnosed within 22 months after surgery. Three cases were diagnosed later (31, 35 and 56 months). A total of 7 complete flap losses (33%) occurred following flap ORN diagnosis. Of these, 6 patients (86%) received a new osseous free flap and 1 (14%) received a new soft tissue free flap in combination with a 3.0 mm load-bearing reconstruction plate. 14 flaps (66%) remained *in situ*. Among these, 4 flaps (29%) were treated with sequestrectomy, iliac crest bone graft and platelet-rich fibrin (PRF) application, 3 (21%) with sequestrectomy and PRF application and 1 (7%) with sequestrectomy and alloplastic coverage. Additionally, 3 patients (21%) required extraoral soft tissue free flaps to achieve sufficient wound closure. 3 flaps (21%) were successfully managed conservatively via curettage and disinfection of necrotic areas (**Figure 2**).

### 3.5 Multivariate analysis

In the multivariate binary logistic regression analysis, smoking, plate exposure and duration of RT were examined for their association with the development of flap ORN. The logistic regression model was statistically significant χ^2^(3) = 15.91, p = 0.001 with a good amount of explained variance (Nagelkerke R^2^ = 0.42), a specificity and sensitivity of each 76.2%. The Hosmer-Lemeshow goodness-of-fit test indicated a good fit (p = 0.64). Smoking (OR 5.78; 95%-CI [1.20;27.92]; p = 0.03) and plate exposure (OR 5.61; 95%-CI [1.18;26.66]; p = 0.03) remained significant predictors of flap ORN. The ROC-AUC was 0.83 (95%-CI [0.70;0.95]), indicating a good ability to distinguish between ORN and no ORN.

## 4 Discussion

While many studies have investigated the role of radiotherapy and specific risk factors in the formation of mandibular ORN (14–18), factors contributing to ORN formation in head and neck osseous free flaps remain largely unknown. This study is the first to assess patient- and surgery-specific predictors of free flap ORN based on a large-scale single-center cohort. To analyze potential predictors in a relatively homogenous cohort, we conducted a matched-pair analysis to control for potential confounders such as gender, age, follow-up time and reconstruction site.

In this study, 19% of all irradiated osseous free flaps developed ORN inside the flap. Of these cases, 33% resulted in complete flap loss, requiring surgical intervention. In most of these cases, additional free flaps, setting the patient at risk for general anesthesia related complications and increased donor site morbidity, were necessary. All other ORN-affected flaps were managed conservatively. Nevertheless, subsequent dental rehabilitation with implants was not achievable in conservatively managed flaps, counteracting one of the main purposes of osseous reconstructions. Therapeutically, conservative management of flap ORN may also benefit from more recently introduced regimes for native bone. For instance, the pentoxifylline-tocopherol-clodronate (PENTOCLO) protocol may be applied for osseous flaps as well and could possibly prevent severe cases (19, 20). Future therapeutical concepts in flap ORN should also account for previously applied radiation doses to optimize surgical outcomes as described before (21, 22). These findings provide both epidemiological and therapeutic insights into free flap ORN. Since the primary goal of reconstructive surgery is to offer patients long-lasting, functional solutions, flap irradiation significantly compromises these outcomes in a substantial number of cases, raising the question about how to prevent such serious consequences.

Previous studies have focused primarily on radiooncologic parameters, whereas this study aimed to also identify patient- and therapy-specific predictors. In both uni- and multivariate analysis, history of smoking emerged as a robust predictor, present in 75% of patients with flap ORN. This association has also been previously described for mandibular ORN formation (16–18). Mechanistically, smoking is known to influence vascularity, promote tissue ischemia and to impair healing processes (23–25), which may contribute to the pathogenesis of ORN in osseous free flaps. Additionally, the adverse effects of smoking are exacerbated by simultaneous alcohol abuse. Although not statistically significant, 52% of patients with flap ORN had a history of alcohol abuse, compared to 33% in the control group. Excessive alcohol consumption has previously been linked to mandibular ORN (26), and its potential role in free flap ORN should not be dismissed. Further studies are needed to assess toxic effects of both substances on ORN formation. These results highlight the need for enhanced risk assessment in patients undergoing postoperative radiotherapy after osseous free flap reconstruction, particularly among smokers. Moreover, smoking is a known risk factor for implant placement. Hence, dental rehabilitation with implants in patients with irradiated flaps and a history of smoking should be questioned (27). Patients with a history of smoking should undergo close monitoring, with frequent follow-up visits focusing on early signs of ORN in osseous flap areas.

In addition to smoking as a vascular risk factor, we also investigated other vascular parameters in this cohort. Anamnestic factors such as atherosclerosis, hyperlipidemia or history of thrombosis did not significantly influence ORN formation. However, the small number of cases must be taken into account. Nevertheless, the role of aberrant fluid dynamics, particularly after radiotherapy, remains a topic of interest. Given radiotherapy’s known vascular damage potential (28, 29) and ORN’s initial vascular etiology (7, 30), the effects of blood flow dynamics warrant further investigation. A previous study has already linked external carotid artery stenosis after radiotherapy with mandibular ORN formation (31). This observation may also be relevant to free flap ORN.

Another surgical aspect examined was the impact of different osteosynthesis plates. A previous study reported two cases of severe free flap ORN adjacent to patient-specific 3D-printed (PS-3D) reconstruction plates (6). Our results did not confirm this association in cohort comparison; however, nearly all patients received PS-3D plates (98%), with the majority receiving PS-3D reconstruction plates (68%). Moreover, prior plate exposure was significantly higher in ORN flaps. If plates and subsequently osseous flap aspects are exposed to the oral microbiome, potential infections and tissue damages occur more likely (32). If the healing capacity is reduced due to prior radiotherapy and even smoking, chances of ORN development could be higher. Additionally, analysis of sub-flap location revealed a refined insight. In fact, all posterior and circular lesions, as well as 50% of anterior lesions occurred next to reconstruction plates. With most of posterior and circular lesions being initially covered by skin paddles but still showing exposed plates in most cases, reconstruction plates may produce a significant amount of scatter radiation or even hyperthermia which subsequently damage covering tissues. Anterior flap aspects are not covered with skin paddles as frequently due to the consequential bulky soft tissue situation. While prior plate exposure occurred in the majority of anterior ORN cases, only 50% occurred in the presence of reconstruction plates, due to the common use of mini plates in this region (33–35). As a result, we assume that irradiation of reconstruction plates may produce more soft tissue damage and leads to plate exposure even though they are mostly covered by skin paddles. Conversely, mini plates could cause lower amounts of scatter irradiation but ORN lesions still occur possibly due to frequent plate exposure following higher biomechanical load in the anterior segment (36) and infrequent skin paddle coverage. The influence of plate geometry and materials on scatter radiation and dose inaccuracy should be further explored through in-silico and experimental studies.

While our results provide evidence of risk factors for free flap ORN, several open questions remain regarding its pathogenesis. Specifically, the influence of dose-toxicity relationship on flap ORN formation remains under debate due to conflicting findings (12). To date, dose-toxicity relationships have only been assessed in osseous aspects of free flaps. This study introduces the potential contribution of osteosynthesis materials, suggesting that their irradiation plays a role in tissue damage and subsequent ORN formation. Further research should also examine the impact of vessel irradiation on ORN formation. Additionally, defining the osseous free flap as structures at risk and delineating the flap should especially be considered in the context of immediate reconstruction, as previously proposed (2, 11, 37). By doing so, the risk of free flap ORN could be drastically reduced, sparing patients from extensive and morbidity-increasing complications, such as unnecessary flap loss and re-transplantation. Furthermore, if osseous flap aspects received no radiation, the study of dose-toxicity relationships and the testing of dose constraints may become unnecessary, as the risk for ORN and subsequent revision surgery would be negligible. While feasibility and prospective studies are needed, close follow-up focusing on flap ORN in at-risk patients is currently recommended.

### 4.1 Limitations

This study has several limitations. First, while case matching allows for cohort homogenization, some patients were excluded from the analysis, potentially leading to underreporting of predictors. Secondly, the small sample size may reduce the generalizability of the results. Third, flap delineation was done *post-hoc* on the initial planning computed tomography, limiting interpretability of radiation-specific results. Lastly, since only one ORN case occurred in the maxilla, our findings mainly apply to mandibular pathologies.

### 4.2 Conclusions

In this single-center retrospective matched-case study, smoking and plate exposure were identified as strong predictors of osseous free flap ORN formation. Additionally, the study provides extensive insights into therapeutic implications of free flap ORN, showing that this side effect of radiotherapy can lead to severe consequences. A significant proportion of affected patients required a new transplant, substantially increasing morbidity. These results emphasize the need for individualized risk assessment and critical evaluation of osseous free flap irradiation in future radiooncological concepts.

## Data Availability

The datasets presented in this article are not readily available because the data are therapy-specific patient data. Requests to access the datasets should be directed to Jakob Fenske, jakob.fenske@charite.de.
